# Hypertensive Havoc: When Malignant Hypertension Mimics Thrombotic Thrombocytopenic Purpura

**DOI:** 10.7759/cureus.101117

**Published:** 2026-01-08

**Authors:** Sarah Kim, Amogh Pathak, Marjan Koch

**Affiliations:** 1 Anesthesiology, Cooper Medical School of Rowan University, Camden, USA; 2 Hematology and Medical Oncology, Cooper University Hospital, Camden, USA

**Keywords:** malignant hypertension (mht), microangiopathic hemolytic anemia (maha), thrombocytopenia, thrombotic microangiopathy, thrombotic thrombocytopenic purpura

## Abstract

Malignant hypertension (HTN) is defined by a rapid rise in blood pressure exceeding 200/130 mmHg, leading to acute end-organ damage. Although relatively uncommon, malignant HTN can cause thrombotic microangiopathy (TMA), characterized by microangiopathic hemolytic anemia, thrombocytopenia, and elevated serum lactate dehydrogenase (LDH). This presentation can mimic thrombotic thrombocytopenic purpura (TTP), a hematologic emergency characterized by thrombocytopenia, hemolytic anemia, renal and neurologic dysfunction, and fever. We present the case of a 28-year-old male patient who presented with seizures and hypertensive emergency. Imaging showed posterior reversible encephalopathy syndrome (PRES), and labs revealed anemia, thrombocytopenia, low haptoglobin, elevated LDH, and schistocytes. A PLASMIC (platelets, lysis, active cancer, stem cell/solid organ transplant, mean corpuscular volume, international normalized ratio, and creatinine) score of 5 indicated intermediate risk for TTP, prompting empiric treatment with corticosteroids and therapeutic plasma exchange (TPE). Platelets normalized, but hemolysis persisted. When clinical reassessment revealed that lab trends correlated with blood pressure fluctuations, TPE and steroids were discontinued, and aggressive antihypertensive therapy led to the resolution of hemolysis. A normal ADAMTS13 level confirmed the diagnosis of malignant HTN-induced TMA. This case underscores the importance of distinguishing malignant HTN from TTP, as their management differs significantly. This case highlights the diagnostic challenge in distinguishing TTP from malignant HTN-induced TMA and underscores the importance of recognizing this rare manifestation of hypertensive crisis to avoid unnecessary therapies and guide appropriate treatment.

## Introduction

Malignant hypertension (HTN) occurs when blood pressure (BP) rises rapidly within a short timeframe, specifically to a BP that is greater than 200/130 mmHg. This can cause acute damage to multiple organs, most commonly the retinas, brain, and kidneys. The pathophysiology involves injury to the vascular endothelium, resulting in increased vascular permeability, fibrinoid necrosis, and ischemic injury to vital organs [1]. Less commonly, malignant HTN can cause thrombotic microangiopathy (TMA), which is the presence of microangiopathic hemolytic anemia, thrombocytopenia, and increased serum lactate dehydrogenase (LDH). Though malignant HTN has a high incidence, affecting as many as 1% of all hypertensive patients, cases where it causes TMA are rare [2]. 

Malignant HTN-induced TMA can therefore be confused with thrombotic thrombocytopenic purpura (TTP), which is characterized by a pentad of fever, thrombocytopenia, hemolytic anemia, renal dysfunction, and neurologic dysfunction. TTP is a hematologic emergency caused by a severe deficiency in ADAMTS13, a von Willebrand factor-cleaving protease. This deficiency leads to the accumulation of large von Willebrand factor multimers, which results in excessive platelet aggregation and microthrombi formation [3].

Given the significant overlap in clinical presentation between malignant HTN-induced TMA and TTP, differentiating between these conditions is crucial for appropriate management. Misdiagnosing malignant HTN-induced TMA as TTP may expose patients to unnecessary therapeutic plasma exchange (TPE), which carries risks, most commonly allergic reactions and electrolyte abnormalities such as hypocalcemia, while delaying definitive BP control, the cornerstone of treatment for malignant HTN-induced TMA. In this report, we present a case of a 28-year-old male patient who presented with seizures and hypertensive emergency. The case highlights the diagnostic challenges and critical differences in management between malignant-HTN induced TMA and TTP.

## Case presentation

Patient presentation and initial evaluation

A 28-year-old male patient with a past medical history of end-stage renal disease on hemodialysis (Monday/Wednesday/Friday), type 2 diabetes mellitus, chronic HTN, and intravenous drug use presented to an outside hospital after two witnessed generalized tonic-clonic seizures lasting approximately 60 seconds each. He was found to be in hypertensive emergency with a BP of 223/131 mmHg and was started on a nicardipine drip.

Due to a persistent headache, non-contrast magnetic resonance imaging of the brain was ordered and demonstrated evidence of posterior reversible encephalopathy syndrome (PRES). Subsequently, he was started on antiepileptic levetiracetam 500 mg twice daily, with an additional 250 mg on dialysis days per neurology recommendations.

Laboratory findings

**Figure 1:**
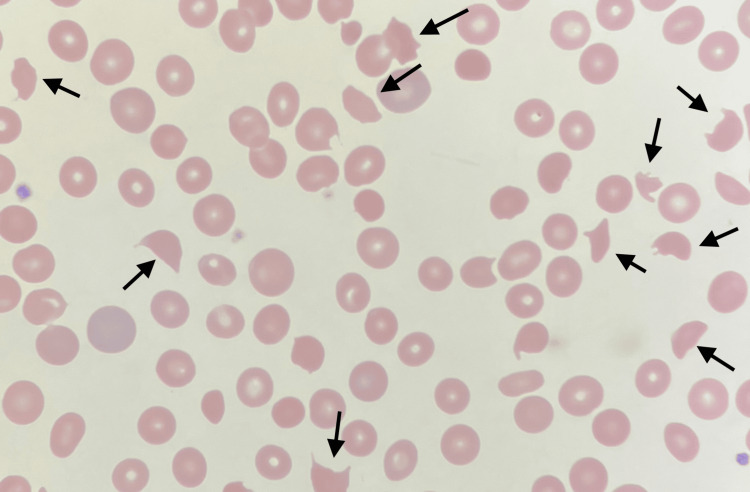
Peripheral blood smear demonstrating multiple schistocytes, indicating microangiopathic hemolytic anemia

Hemogram revealed hemoglobin of 6.7 g/dL and a platelet count of 42,000/µL. There was no clinical evidence of gastrointestinal bleeding. Evidence of hemolysis, including low haptoglobin (<10 mg/dL), elevated LDH (742 U/L), and schistocytes on peripheral smear (Figure 1), in addition to the recent initiation of hemodialysis for progressive renal disease raised suspicion for TTP. The patient had a PLASMIC (platelets, lysis, active cancer, stem cell/solid organ transplant, mean corpuscular volume, international normalized ratio, and creatinine) score of 5, indicating an intermediate risk of TTP and a 6% chance of severe ADAMTS13 deficiency. He was subsequently transferred to a tertiary center for escalation of care.

Initial treatment and clinical course

**Figure 2:**
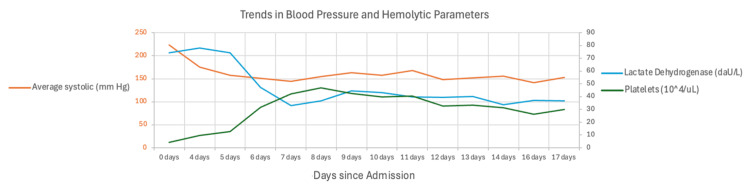
Graph demonstrating the relationship between blood pressure control and platelet/hemolytic anemia parameters over time

Upon transfer to the higher-level center (day 4 of Figure 2), the patient was started on high-dose prednisone and underwent two sessions of TPE. Platelet counts had improved before initiating this treatment for TPE. After two days, they had normalized; however, hemolytic parameters continued to worsen, along with evidence of persistent schistocytes.

Diagnostic reevaluation and final diagnosis

Platelet drop, as well as improvement, was correlated to the patient's BP readings (Figure 2, Appendices). After reevaluation, it was evident that the patient's schistocytes were a result of malignant HTN. Steroids and TPE were discontinued. Elevated BPs were aggressively managed with a four-drug regimen consisting of nifedipine 90 mg daily, labetalol 400 mg three times a day, hydralazine 100 mg three times a day, and torsemide 100 mg daily. BP control was further supported with intermittent aggressive ultrafiltration during his scheduled hemodialysis sessions. Hemolytic parameters subsequently improved. An ADAMTS13 level that was sent the day the patient transferred to the higher center was eventually found to be normal, confirming the exclusion of TTP and proving malignant HTN-induced TMA as the final diagnosis.

The patient was discharged 23 days after the initial presentation and returned to the care of a state prison.

## Discussion

Pathophysiology and diagnostic overlap of malignant HTN and TTP

**Figure 3:**
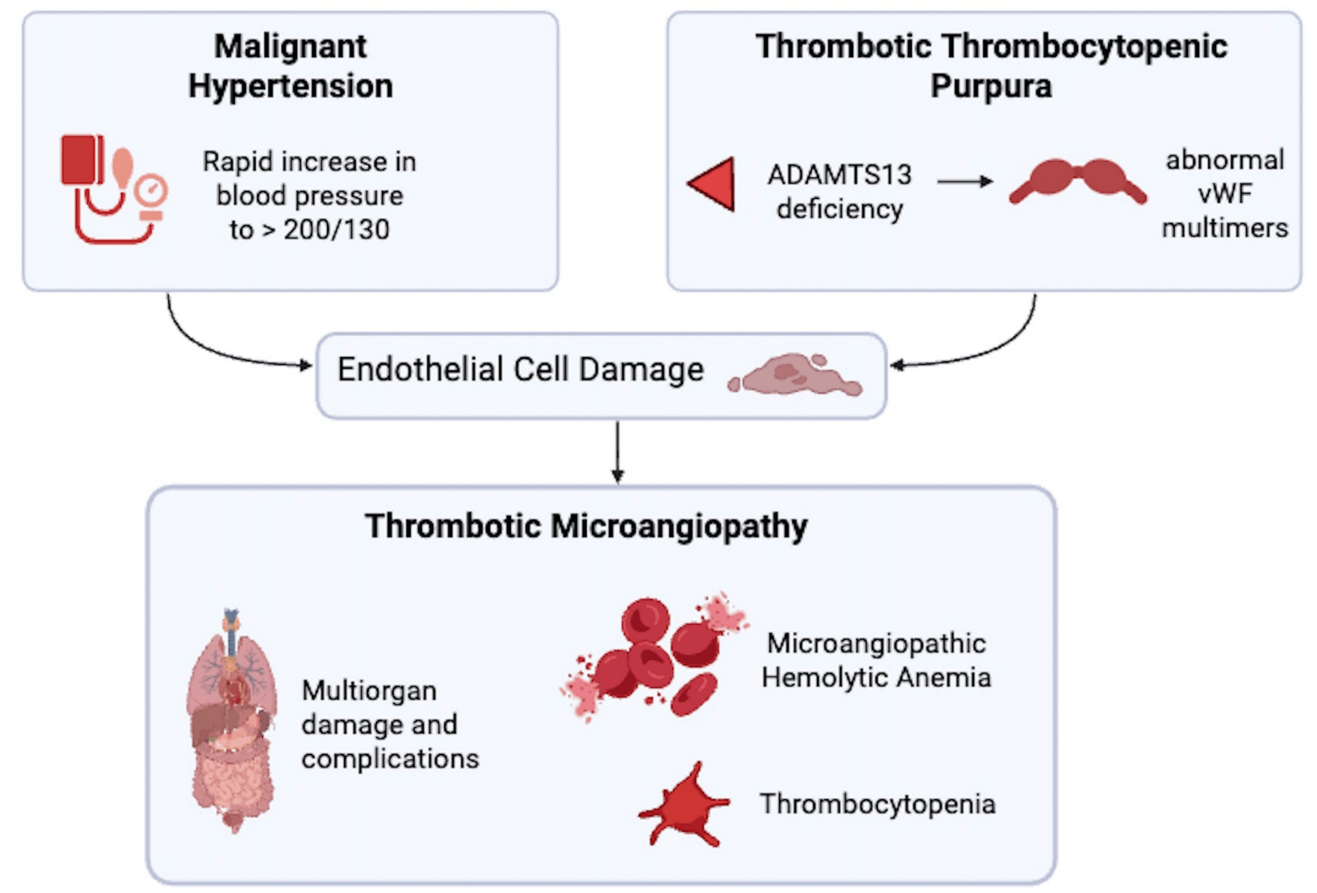
Malignant hypertension and thrombotic thrombocytopenic purpura, though distinct pathologies, can both lead to thrombotic microangiopathy (created with Biorender.com) vWF: von Willebrand factor

This case illustrates the diagnostic challenge in distinguishing malignant HTN-induced TMA from TTP (Figure 3). TMA is an umbrella term that encompasses a wide range of pathologic processes, characterized by microangiopathic hemolytic anemia (MAHA), thrombocytopenia, and microthrombi leading to end-organ ischemic damage [4]. Clinically, a number of conditions present with MAHA and thrombocytopenia, some of which can be life-threatening (e.g., TTP/hemolytic uremic syndrome (HUS)). The differential for the underlying cause is broad and includes conditions like cancer, infection, solid organ transplant, drug use, autoimmune diseases, and preeclampsia/HELLP (hemolysis, elevated liver enzymes, and low platelet count) syndrome [5].

Malignant HTN-induced TMA is a form of secondary TMA, meaning that it is TMA occurring in the context of another disease process [4]. Our patient presented with a BP of 223/131 mmHg and was therefore considered to be severely hypertensive [1]. Even so, TMA does not always occur in the setting of malignant HTN, instead being noted to occur 14-47% of the time [6]. When malignant HTN-induced TMA does occur, the rapid, extreme, and sustained elevation in BP directly causes damage to endothelial cells, causing leakage of plasma components into the vascular wall and subsequent fibrinoid necrosis, particularly in high-resistance circulatory beds like the kidney, brain, and retina [7]. This further leads to the characteristics of TMA: microangiopathic hemolytic anemia, consumptive thrombocytopenia, and microthrombi formation [6].

TTP is a form of primary TMA, meaning that it is TMA that occurs spontaneously without a clear associated underlying cause [4]. TTP occurs when ADAMTS13, a metalloprotease responsible for breaking down large von Willebrand factor multimers into smaller fragments, is severely deficient, specifically to <10%. These deficiencies can be either acquired due to autoantibody formation against ADAMTS13 or inherited due to a mutation in the ADAMTS13 gene. When not enough ADAMTS13 is present, unusually large von Willebrand factor multimers in the plasma bind to platelets, leading to the unregulated activation of platelets and resultant microthrombi formation. Clinically, in addition to fever, thrombocytopenia, and hemolytic anemia, the classic TTP pentad also consists of neurologic and renal dysfunction [3,4,8-13]. In this way, our patient's initial presentation of seizures and radiologic evidence of PRESS, as well as his worsening renal function and recent initiation of dialysis, led to a high suspicion for TTP and initial treatment with steroids and TPE.

Although the pathophysiologies of malignant HTN-induced TMA and TTP differ, they both ultimately lead to endothelial cell damage and ischemic injury to multiple potential organs, most commonly the brain, heart, and kidneys, and contain significant diagnostic overlap. However, it is important to distinguish between these two potentially life-threatening conditions as they necessitate different treatments: TTP requires corticosteroids and emergent plasma exchange, whereas malignant HTN-induced TMA is managed with aggressive BP control. While the most obvious distinguishing factor would be the presence or absence of malignant HTN, in the case where malignant HTN is present, more subtle differences can be noted in the degree of thrombocytopenia and renal impairment. Malignant HTN-induced TMA has been noted to have a lesser degree of thrombocytopenia but a higher degree of renal impairment, determined by creatinine level, in comparison to patients with TTP [14]. However, while these factors can be used in support of a diagnosis, they are not sufficient in confirming one or the other.

Reassessing the diagnosis

Prompt recognition of TTP is critical, as it underpins the rationale for plasma exchange (to replenish ADAMTS13 and remove autoantibodies) and immunosuppression (to reduce antibody production) as the mainstays of therapy [8]. The gold standard for the diagnosis of TTP is ADAMTS13 activity measurement, which often has a significant processing time. When clinical suspicion for TTP is high, treatment is initiated with steroids and therapeutic plasma exchange without awaiting ADAMTS13 results [13]. During this interim period, the PLASMIC score is used to predict the likelihood of significant ADAMTS13 deficiency, incorporating platelet count, hemolysis markers, creatinine, international normalized ratio (INR), mean corpuscular volume (MCV), and history of cancer or transplant into the calculation [15]. Our patient had a PLASMIC score of 5, indicating an intermediate risk for severe ADAMTS13 deficiency, which justified the initial empiric treatment for presumed TTP.

However, several clinical features warranted the reconsideration of this diagnosis. Most notably, platelet count improved prior to the initiation of TPE. Even after TPE initiation (day 4 of Figure 2), platelets continued to improve and normalized within two days, while markers of hemolysis (e.g., LDH, schistocytes) continued to worsen. If TTP were truly the correct diagnosis, these hemolytic parameters would be expected to demonstrate rapid improvement, particularly given that our patient underwent two sessions of therapeutic plasma exchange. Instead, a retrospective analysis of the diagnosis revealed a striking correlation between BP fluctuations and dynamic changes in platelet and hemodynamic parameters. With further control of the patient's BP, platelets remained stable and hemolytic markers eventually improved, pointing to malignant HTN as the primary cause of his TMA. Moreover, our patient's ADAMTS13 level eventually returned to normal, ultimately confirming the exclusion of TTP.

## Conclusions

We want to use this case to highlight that TMA has a broad differential diagnosis and to underscore the importance of dynamic clinical reassessment. When clinical suspicion of TTP is high, such as due to a high PLASMIC score, initial empiric treatment with TPE is typically appropriate given the urgency of treatment in TTP. However, when laboratory or clinical response deviates from the expected course of TTP, such as ongoing hemolysis despite platelet recovery, alternative diagnoses should be revisited in order to avoid overtreatment and guide appropriate care. Inappropriate or prolonged plasma exchange exposure may increase the risk of infections, transfusion reactions, and other complications. In this case, tapering and eventual discontinuation of TPE and corticosteroids, alongside intensified antihypertensive therapy, led to the resolution of hemolytic parameters and confirmation of the diagnosis of malignant HTN-induced TMA.

## Appendices

**Table 1:** Trends in blood pressure and hemolytic parameters Table corresponding with Figure 2, demonstrating the relationship between blood pressure control and platelet/hemolytic anemia parameters over time.

Days since admission	Average systolic (mmHg)	Platelets (10^4^/uL)	Lactate dehydrogenase (U/L)
0 days	223	4	74.2
4 days	175	10	78.1
5 days	158	13	74.2
6 days	151	32	47.3
7 days	144	42	33.1
8 days	155	47	36.8
9 days	163	43	44.5
10 days	158	39.9	43.2
11 days	168	40.6	39.9
12 days	148	32.7	39.5
13 days	152	33.3	40
14 days	156	31.2	33.8
16 days	142	26.1	37.1
17 days	153	29.8	36.8

## References

[1] Tsige AW, Ayele SG (2024). Malignant hypertension: current challenges, prevention strategies, and future perspectives. Front Cardiovasc Med.

[2] Khanal N, Dahal S, Upadhyay S, Bhatt VR, Bierman PJ (2015). Differentiating malignant hypertension-induced thrombotic microangiopathy from thrombotic thrombocytopenic purpura. Ther Adv Hematol.

[3] Stanley M, Killeen RB, Michalski JM (2025). Thrombotic thrombocytopenic purpura. StatPearls [Internet].

[4] Arnold DM, Patriquin CJ, Nazy I (2017). Thrombotic microangiopathies: a general approach to diagnosis and management. CMAJ.

[5] Scully M, Cataland S, Coppo P (2017). Consensus on the standardization of terminology in thrombotic thrombocytopenic purpura and related thrombotic microangiopathies. J Thromb Haemost.

[6] Cavero T, Auñón P, Caravaca-Fontán F (2023). Thrombotic microangiopathy in patients with malignant hypertension. Nephrol Dial Transplant.

[7] van den Born BJ, Honnebier UP, Koopmans RP, van Montfrans GA (2005). Microangiopathic hemolysis and renal failure in malignant hypertension. Hypertension.

[8] Sadler JE (2017). Pathophysiology of thrombotic thrombocytopenic purpura. Blood.

[9] Joly BS, Coppo P, Veyradier A (2017). Thrombotic thrombocytopenic purpura. Blood.

[10] Saha M, McDaniel JK, Zheng XL (2017). Thrombotic thrombocytopenic purpura: pathogenesis, diagnosis and potential novel therapeutics. J Thromb Haemost.

[11] Kremer Hovinga JA, Coppo P, Lämmle B, Moake JL, Miyata T, Vanhoorelbeke K (2017). Thrombotic thrombocytopenic purpura. Nat Rev Dis Primers.

[12] Sukumar S, Lämmle B, Cataland SR (2021). Thrombotic thrombocytopenic purpura: pathophysiology, diagnosis, and management. J Clin Med.

[13] Pishko AM, Li A, Cuker A (2025). Immune thrombotic thrombocytopenic purpura: a review. JAMA.

[14] Song Y, Lee SY, Chee YL, Jen WY (2024). Hypertensive emergency with thrombotic microangiopathy or TTP? A case series and literature review. J Clin Med.

[15] Bendapudi PK, Hurwitz S, Fry A (2017). Derivation and external validation of the PLASMIC score for rapid assessment of adults with thrombotic microangiopathies: a cohort study. Lancet Haematol.

